# Feasibility of Backscatter Communication Using LoRAWAN Signals for Deep Implanted Devices and Wearable Applications

**DOI:** 10.3390/s20216342

**Published:** 2020-11-06

**Authors:** Marc Lazaro, Antonio Lazaro, Ramon Villarino

**Affiliations:** Department of Electronics, Electrics and Automatic Control Engineering, Rovira i Virgili University, 43007 Tarragona, Spain; marc.lazaro@urv.cat (M.L.); ramon.villarino@urv.cat (R.V.)

**Keywords:** LoRa, backscatter, implant, antenna, wearable, body area networks

## Abstract

This paper presents a method for low data rate transmission for devices implanted in the body using backscattered Long Range (LoRa) signals. The method uses an antenna loaded with a switch that changes between two load impedances at the rate of a modulating oscillator. Consequently, the LoRa signal transmitted by a LoRa node is reflected in the adjacent channels and can be detected with a LoRa gateway tuned to the shifted channels. A prototype developed to operate at Medical Implant Communication Service (MICS) and the Industrial Scientific and Medical (ISM) 433 MHz band is presented. The prototype uses a commercial ceramic antenna with a matched network tuned to the frequency band with high radiation efficiency. The effect of the coating material covering the antenna was studied. Simulated and experimental results using a phantom show that it is feasible to read data from deep implanted devices placed a few meters from the body because of the high sensitivity of commercial LoRa receivers.

## 1. Introduction

Long Range Wide Area Network (LoRaWAN) is one of the best wireless technologies for the Internet of Things (IoT) [1,2]. Its popularity is due to the robust modulation scheme based on chirp spread spectrum (CSS) modulation [3,4,5]. LoRa receivers exhibit an impressive sensitivity which, depending on the spread factor used, can be as low as −148 dBm (Semtech SX1276 [6]). As a result of this sensitivity, radio links of several kilometers long can be designed [7]. LoRa also provides advantages in both blocking and selectivity and can achieve high interference immunity with low energy consumption and low-cost oscillators. LoRa is based on a low power modulation designed to send small data packages over long distances, operating on a battery. It can demodulate signals which are 7.5 dB to 20 dB below the noise floor (see Table 13 in [6].

The performance of wearable and implantable devices is often limited by the lifetime of batteries. A considerable part of the power budget of these devices is spent by the radiofrequency circuits (oscillators, receivers, and transmitters) on wireless communication [8]. Recently, backscatter communications have been raising a great interest in a variety of applications including implanted devices [9,10,11,12]. Backscatter communication requires zero transmission power and works by backscattering the RF signal sent by an external transmitter [13,14]. This type of communication is used in Radio Frequency Identification (RFID) systems. Various backscattering systems compatible with commercial wireless transmitter signals have been proposed in the literature [15,16,17]. The simplest backscatters (see [14] for a survey of ambient communications) generate On-Off Keying (OOK) or Frequency Shift Keying (FSK) signals from an unmodulated carrier and are encoded by a custom receiver (often an Software Defined Radio (SDR)). However, recently, backscattering systems based on complex modulations such as Bluetooth, Zigbee, or WiFi have been reported [14]. In [17], a frequency-shifted backscatter was described where the backscatter tags shift the carrier signal to an adjacent frequency band using WiFi and Bluetooth devices for the carrier generation and receiver. In [12], a Bluetooth transmitter is used to generate packets with a backscatter on WiFi or ZigBee channels that modifies the Received Signal Strength Indicator (RSSI) of the WiFi or Zigbee receiver. However, in the latest approach an Field-Programmable Gate Array (FPGA) platform with high power consumption was used to generate the WiFi or Zigbee backscatter packets. A similar approach has been proposed to generate LoRa compatible chirps using an FPGA [18,19]. This approach also requires a custom receiver based on expensive SDR [18,19] and increases the data throughput at the expense of prohibitive power consumption for implanted medical devices (almost using an FPGA as a replacement for a custom IC, which theoretically would consume significantly less).

In this paper, we discuss a novel communication method based on a frequency-shifted LoRa backscatter for wearable and deep implanted devices using Medical Implant Communication Service (MICS) bands and the ISM 433 MHz band. The system is designed for low-data rate transmission and is based on the detection of LoRa packets backscattered at the adjacent channels. A standard commercial LoRa node and LoRa gateway are used as transmitter and receiver, respectively, but the gateway receives packets at the frequency-shifted channel. Long-distance links can be achieved using this approach in the air [18,19]. Therefore, the communication based on LoRa backscatter can be useful for communication with deep implants. The strong attenuation of the body is compensated by the sensitivity of the LoRa receivers. To this end, the backscatter must be connected to a specifically designed implanted antenna. The miniaturization of the implanted antenna decreases the radiation efficiency, but this is mitigated by the sensitivity of the system. This paper uses off the shelf components to design a specific antenna, which is tuned to the desired frequency adjusting the matching network and covering it with a heat shrinkable sleeve.

The paper is organized as follows. Section 2 describes the theory of communication based on the backscatter LoRa signal. A proof of concept is provided for the implanted tag. Fundamental to the miniaturization of the tag is the design of a miniature antenna especially tuned to the 406–433 MHz bands. The phantom used in the experiments is characterized. Section 3 presents experimental results with the proposed system. Section 4 compares the system with other approaches reported in the literature. Finally, Section 5 draws the conclusions.

## 2. Materials and Methods

### 2.1. System Description and Theory of Operation

**Figure 1:**
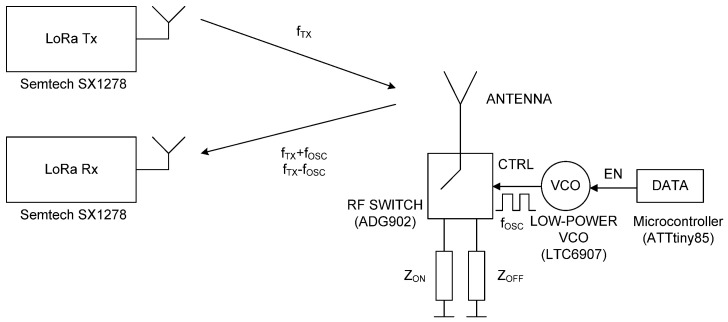
Block diagram of the backscattering system.

The system consists of a zero power backscatter, a LoRa transmitter node, and a LoRa receiver (gateway). Commercial transceivers are used as both transmitter and receiver. Therefore, the system does not require the reader to be specifically developed, only tuned to the desired frequencies and with antennas for the desired bands. This may make it easier to develop implanted devices because there is no need for custom developments. A block diagram of the system is shown in Figure 1. In order to maximize the backscattered power, the backscatter consists of an antenna connected to an RF switch that loads the antenna and switches between two high reflection coefficient states (e.g., open circuit and short circuit). The antenna is tuned to the Medical Implant Communication Service (MICS) band (401–406 MHz) [20], although it can also be tuned to other bands such as the Industrial Scientific and Medical (ISM) 433 MHz band, which is also frequently used for Implanted Medical Devices (IMD). The switch is controlled by a low-power square wave oscillator with oscillation frequency *f_osc_*.

The backscattered field of an antenna ${\vec{E}}_{S}$ is a function of the antenna load *Z_L_*. It can be split as a sum of two terms [21,22]:

(1) $${\vec{E}}_{S}\left(Z_{L}\right)={\vec{E}}_{est}+{\vec{E}}_{m}\Gamma$$

The first term is the structural mode ${\vec{E}}_{est}$, which is independent of the load, and the second (${\vec{E}}_{m}$) is the antenna mode (also known as tag mode). ${\vec{E}}_{est}$ is the scattered field when the tag is connected to a reference load *Z_L_* = Z_a_*, where *Z_a_* is the antenna’s impedance. Γ is the power reflection coefficient given by [23]:

(2) $$\Gamma =\frac{Z_{L}-Z_{a}^{*}}{Z_{L}+Z_{a}}$$

When the load is the conjugate impedance of the antenna impedance, all the incident power is transferred to the load and the antenna only reflects the structural mode. The structural mode arises from the current induced on the antenna’s conducting surface by the incident wave. It does not depend on the load but depends on characteristics such as antenna type, geometry, and material. Thus, the structural mode is independent of the load reflection coefficient, whereas the antenna mode is proportional to it.

The reflection coefficient can be modulated by switching the antenna load between two impedance states (*Z_ON_*, *Z_OFF_*). As a first approximation, the reflection coefficient can be approximated by a square waveform with amplitude ΔΓ = Γ_ON_ − Γ_OFF_. Therefore, this periodic signal with period 1/*f_osc_* can be developed in a Fourier series and the spectrum is given as an infinite sum of delta functions located at the harmonics:

(3) $$\Gamma \left(f\right)=\overset{n=+\infty }{\underset{n=-\infty }{\sum }}c_{n}\delta \left(f-\left(f_{c}+nf_{osc}\right)\right)+\overset{n=+\infty }{\underset{n=-\infty }{\sum }}c_{n}\delta \left(f+\left(f_{c}+nf_{osc}\right)\right)$$

where *c_n_* are the Fourier coefficients, and *f_c_* is the input frequency of the incident signal that illuminates the tag. For a square waveform with duty cycle *δ_c_*, the coefficients *c_n_* are given by:

(4) $$c_{n}=\{\begin{array}{cc} \frac{1}{2}\Gamma_{avg} & ,n=0\\ \frac{1}{2}\left|\Delta \Gamma \right|\delta_{c}\left(\frac{\sin\left(n\pi \delta_{c}\right)}{n\pi \delta_{c}}\right) & ,n\neq 0 \end{array}$$

where Γ*_avg_* is the average power reflection coefficient between ON and OFF states.

Using (3), the backscattered field can be written as:

(5) $$\begin{array}{c} {\vec{E}}_{S}\left(Z_{L}\right)=\left({\vec{E}}_{S}+\frac{1}{2}{\vec{E}}_{m}\Gamma_{avg}\right)\left(\delta \left(f-f_{c}\right)+\delta \left(f-f_{c}\right)\right)+{\vec{E}}_{m}\sum_{n\neq 0}c_{n}\delta \left(f-\left(f_{c}+nf_{osc}\right)\right)\\ +{\vec{E}}_{m}\sum_{n\neq 0}c_{n}\delta \left(f+\left(f_{c}+nf_{osc}\right)\right) \end{array}$$

Therefore, the first term in (5) results in the non-modulated term and depends on the structural mode of the tag. The second term represents the modulated side-bands that are a function of the antenna mode. Coefficients of higher amplitude in the Fourier expansion (n = ±1), are which result in the components at the frequencies *f_c_* ± *f_osc_*. 

**Figure 2:**
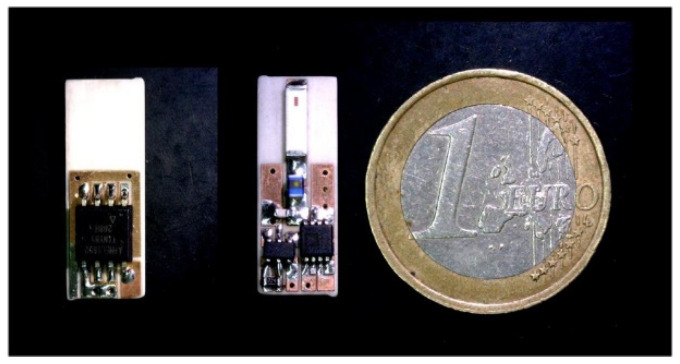
Photograph of LoRa bottom backscatter. Microcontroller and top layer with the RF switch and oscillator.

The result is that the LoRa channel at transmission frequency *f_TX_* is also reflected in the adjacent channels with central frequencies given by *f_TX_* ± *f_osc_*. Therefore, a LoRa receiver tuned to the frequency of one of these shifted channels at *f_TX_* ± *f_osc_* can demodulate the transmitted packed if the backscatter is enabled. Enabling and disabling the modulating oscillator can transmit low-rate data for telemetry purposes. LoRa gateways are intermediaries that allow sensing devices to transmit data to the cloud. Modern LoRa gateways are based on chipsets that can simultaneously receive LoRa messages on multiple frequency channels. Therefore, it is possible to demodulate the signal shifted by the backscatter to another frequency channel. The proof of concept uses the ADG902 (Analog Devices Inc., Norwood, MA, USA) CMOS switch [24] and the LTC6907 (Analog Devices Inc., Norwood, MA, USA) [25] oscillator, and the 0900AT43A0070 antenna (Johanson Technology, Camarillo, CA, USA) [26], which can be tuned to the desired frequency with some adjustments. LTC6907 is a low-power clock generator for which the oscillator frequency *f_osc_* can be adjusted to between 40 kHz and 4 MHz with a resistor that has a current consumption between 36–100 µA at 400 kHz to 1 MHz. The LTC6907 has 1% frequency accuracy from 0 °C to 70 °C, which is enough for this application. Figure 2 shows the system integrated into a small-size PCB. In addition, the LoRa receiver can measure the frequency offset in the received packet and therefore can be adjusted to minimize the effects of oscillator drifts. The experiments use the Semtech SX1278 LoRa transceiver (Semtech Corp., Camarillo, CA, USA), which can work from 137 MHz to 525 MHz and cover the MICS and ISM 433 MHz bands.

**Figure 3:**
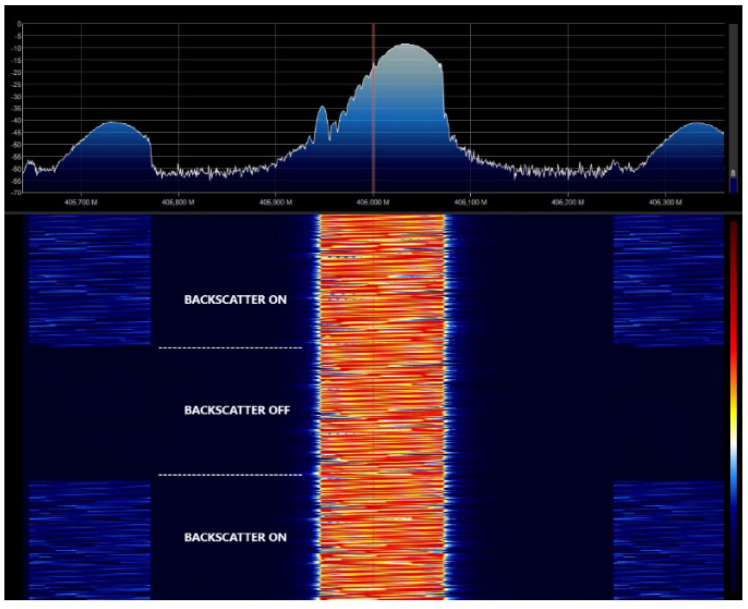
Measured spectrogram with the backscatter enabled and disabled for *SF* = 12, *BW* = 125 kHz.

In order to show the modulation of LoRa packets, Figure 3 shows the measured spectrogram (spectrum as a function of the time) of the modulated LoRa channel at 406 MHz continuously sending packets (with a spread factor of 12 and a bandwidth of 125 kHz) with the backscatter enabled (and the modulating oscillator enabled) and disabled. The measurement was made with an RTL-SDR software defined radio (SDR) connected to a monopole antenna using SDR Sharp software 1 m from the backscatter. The central channel is the transmitted channel, but when the backscatter is enabled, it reflects the signal shifted from the central carrier to the oscillation frequency (*f_osc_* = 300 kHz in this experiment).

The modulated backscattered power at the sideband is proportional to the differential radar cross-section *RCS_dif_* given by [23]:

(6) $$RCS_{dif}=\frac{\lambda^{2}}{4\pi }G_{a}^{2}{\left|\Delta \Gamma \right|}^{2}m$$

where *λ* is the wavelength, *G_a_* is the antenna gain, and *m* is a modulating factor that can be obtained from (4) as *m* = 4|c_1_|^2^/|ΔΓ|^2^ [27]. Assuming an ideal 50% duty cycle and a modulating square wave, *m* is 1/π^2^.

**Figure 4:**
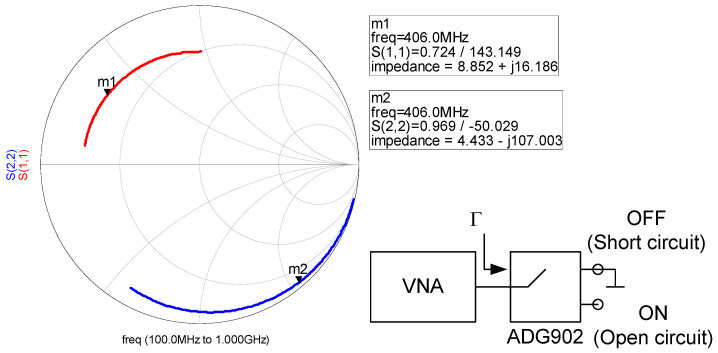
Measured reflection coefficients of the ADG902 switch (S11 state OFF, S22 state ON) as a function of the frequency between 100–1000 MHz.

The backscattered power can be maximized if the antenna resonates in the band (Im*Z_a_* = 0) and the two reflection coefficients are out of phase (e.g., close to short circuit and open circuit) with a phase difference of 180°. In order to obtain the difference in the reflection coefficients (assuming that the antenna is well-matched to the reference impedance 50 Ω) between the two states ΔΓ, a prototype consisting of an SMA connector connected to the ADG902 switch is measured with the Vector Network Analyzer (VNA). Figure 4 shows the measured reflection coefficient when the switch is on the OFF state, which is loaded with a short circuit, and for the ON state, which is loaded with an open circuit. A marker at 406 MHz shows that the two states have a phase difference close to 180°. A loss of 1.5 dB with respect to the ideal short circuit and open circuit case is obtained due to the losses introduced by the switch. 

### 2.2. Data Transmission

**Figure 5:**
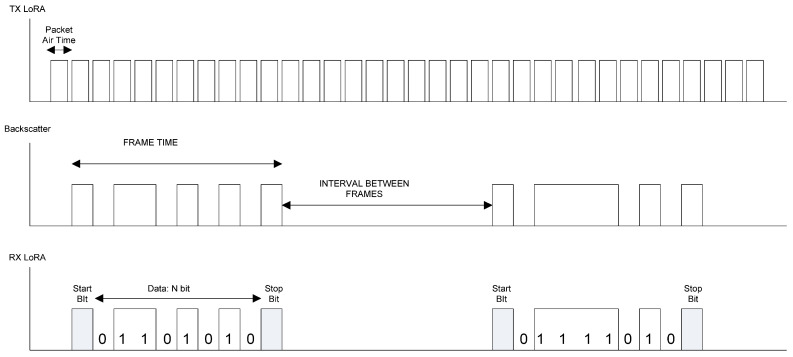
Block diagram showing the data transmission using the reflection of the transmitted packets.

The listen-before-talk approach is used to select a free channel. The LoRa gateway is configured to listen on the LoRa shifted channel. The procedure for data transmission is schematically shown in Figure 5. The protocol is inspired in the well-known asynchronous serial port protocol. The LoRa transmitter node is continuously sending packets, the duration of which (packet air time) depends on the spread factor selected and the number of bits per packet (see Section 2.5). For example, for a packet with SF = 12 and 10 bits containing only the preamble data, its duration is 1 s (considering a small tolerance). The data are encoded in frames. Each frame consists of a start bit and N data bits with the sensor information and ends with a Stop bit. The backscatter reflects the packets enabling the internal oscillator. When the receiver in the gateway receives a packet corresponding to the start bit, it starts to decode the message after an interval between frames. Note that it does not need to receive a complete packet, it is only necessary to detect the presence of a packet. Therefore, it is enough to receive part of the preamble section of the packet. This feature simplifies the synchronization. The LoRa receiver can measure the frequency shift between the expected central frequency and the central frequency measured by the receiver. Therefore, small frequency offsets due to drift in the backscatter oscillator can be dynamically corrected between frames. The frame time is determined from the number of bits per frame (start, payload, and stop bits) multiplied by the interval between packets. Therefore, the frame time depends on the number of bits that will be sent in the application. It can be fixed or variable. In this last case, it is recommended to send some field indicating the total length of the frame. Additional protection such as parity bits, checksums, or CRC bits can be encoded within the data field. The implanted backscatter is assumed to update data cyclically. After the frame transmission, the backscatter changes to sleep mode to reduce power consumption. The backscatter initiates a new frame transmission after a period that is longer than the minimum interval between frames that must be a minimum of a bit duration. However, longer intervals between frames or transmission bursts help to increase the battery life.

### 2.3. Phantom Design

**Figure 6:**
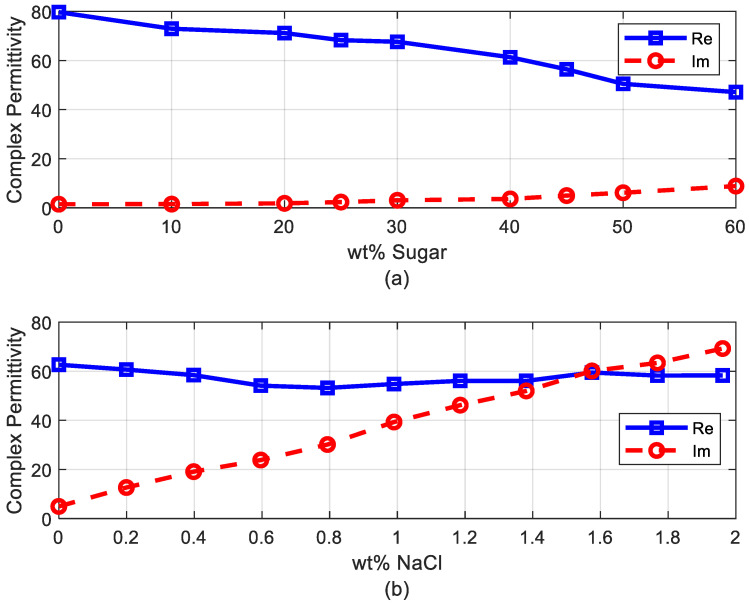
(**a**) Measured dielectric permittivity (real and imaginary part) as a function of sugar mass fraction at 406 MHz, and (**b**) measured dielectric permittivity (real and imaginary part) as a function of NaCl mass fraction and 45% sugar at 406 MHz.

**Figure 7:**
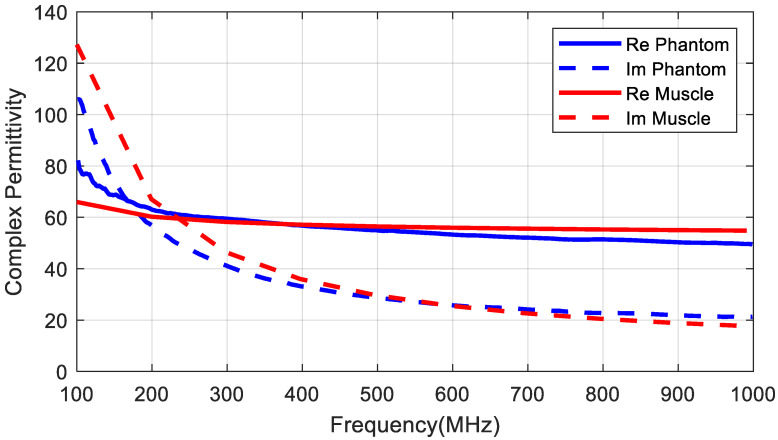
Measured dielectric permittivity (real and imaginary part) as a function of frequency and comparisons with muscle tissue data.

In order to develop an antenna for a deep implant, a phantom material needs to be designed to simulate the muscle tissue for the desired frequency range. The dielectric properties of different tissues have been reported in the literature [28]. To this end, several phantom materials have been proposed for different frequency ranges and applications [29,30,31,32]. In our case, the dielectric properties must be designed to take into account the shift in the antenna frequency response introduced by the high dielectric constant of the tissues and to simulate the attenuation of the body and the reduction in the radiation efficiency of the antenna due to the high tissue conductivity. In our case, we are interested in finding a simple phantom for the UHF band (400–433 MHz). The dielectric constant of the muscle is about 57 and the conductivity is 0.8 at 406 MHz according to data provided by Gabriel et al. [28]. For frequencies higher than 100 MHz, the dielectric constant of the muscle is lower than that of water [30] so a phantom based on a saline water solution is considered because antennas can be used in it more easily than in a solid phantom and it is easy to manufacture. In [31], a mixture of water with NaCl and sucrose was proposed to design a phantom at 2.45 GHz. The present study follows a similar procedure but adjusts the parameters to the 400–500 MHz band. The permittivity was measured using an open-ended coaxial probe [33] from Keysight (slim form probe 85070E/N1501A model, Keysight Technologies, Santa Rosa, CA, USA) with a Vector Network Analyzer (VNA) model Keysight PNA E8364C. Figure 6a shows the real part and imaginary part of the permittivity as a function of the mass fraction (percentage by weight) of sugar at 406 MHz. The imaginary part remains nearly constant whereas the real part decreases as a function of sugar. It can be shown that for a percentage of about 45% of sugar, the real part is close to the muscle value. Figure 6b shows the complex permittivity for a saline water solution with 45% of sugar and changing the quantity of NaCl at 406 MHz. Therefore, adding NaCl can increase the conductivity and adding sugar can reduce the real part. A solution consisting of 45% sucrose, 0.8% NaCl, and water (percentages are in weight) was chosen. Figure 7 compares the data reported by Gabriel et al. [28] and our measurements at ambient temperature (25 °C). Agreement was good in the 400–600 MHz band. The power transported by the wave decreases exponentially in accordance with Lambert’s law. The attenuation constant introduced by the tissue can be computed using [34]:

(7) $$\alpha \left(\frac{dB}{m}\right)=8.686\cdot 2\pi f\sqrt{\frac{\mu_{0}\varepsilon_{0}\varepsilon {}^{\prime }}{2}\left[\sqrt{1+{\left(\frac{\varepsilon {}^{\prime \prime }}{\varepsilon {}^{\prime }}\right)}^{2}}-1\right]}$$

where *ε_c_* = *ε′* − *jε″* is the complex relative permittivity, *ε*_0_ and *µ*_0_ are the vacuum permittivity and permeability, respectively, and *f* is the frequency. Considering the muscle complex permittivity, a typical attenuation of 165 dB/m or a power penetration depth of *d_p_* = 1/(2α(Np/m)) = 2.6 cm is introduced by the muscle at 406 MHz. Equation (7) was derived for incident plane waves in large bodies without considering reflections within the materials at the opposite interface with the air. Ayappa et al. [34] have shown that the above relationship only applies if the thickness of the tissues is larger than the critical length, which is about 5.4*d_p_*. Otherwise, computer simulation models are required to predict more realistic power distributions.

### 2.4. Antenna for Deeply Implanted Backscatter

**Figure 8:**
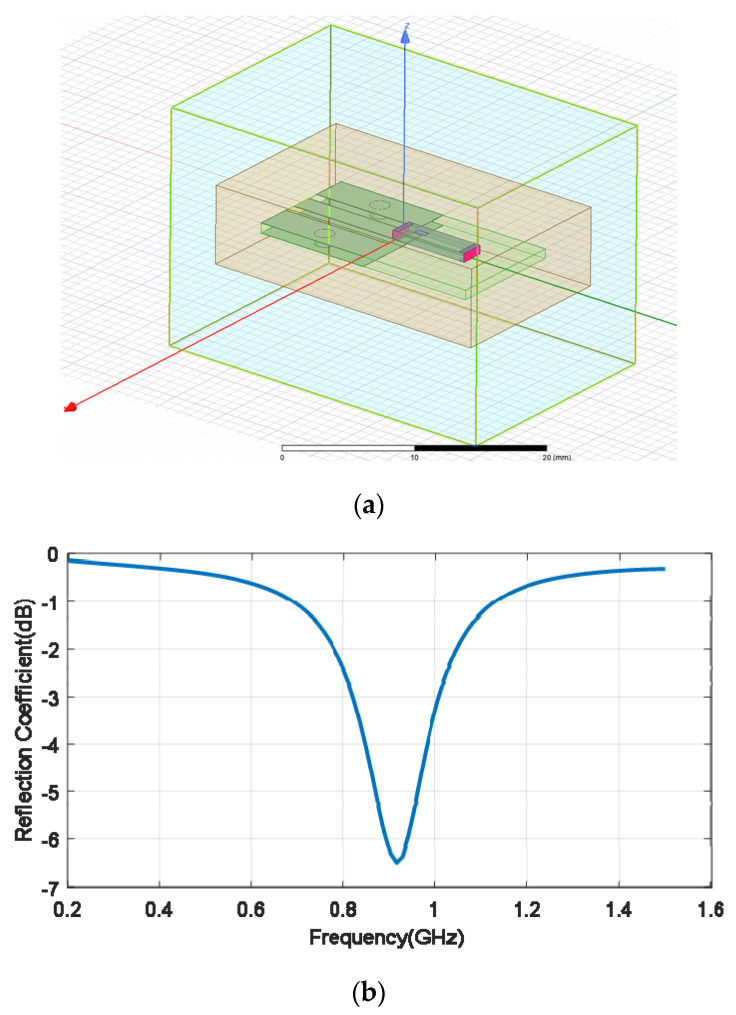
(**a**) Image of the simulated antenna with HFSS (high-frequency structure simulator), (**b**) simulated antenna reflection coefficient as a function of the frequency for the antenna with a 1-mm thick silicone coating.

**Figure 9:**
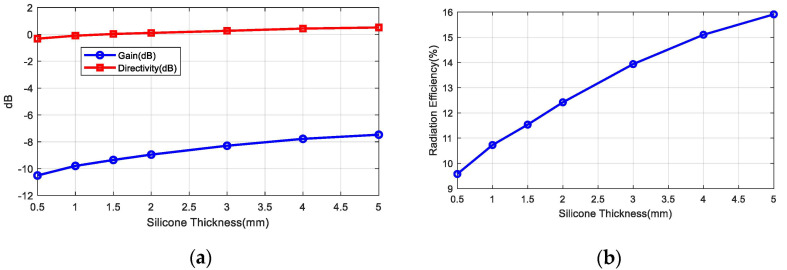
(**a**) Simulated gain and directivity at θ = 0°, ϕ = 0°, and (**b**) radiation efficiency as a function of the silicone thickness at 406 MHz.

**Figure 10:**
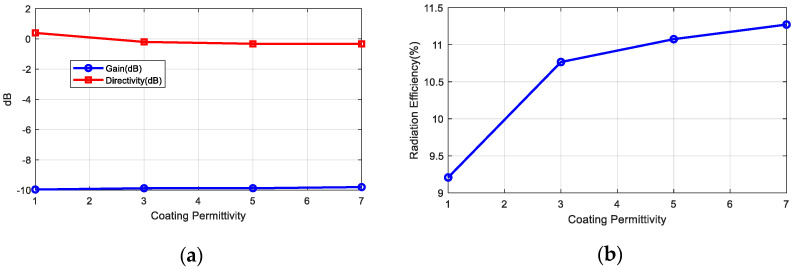
(**a**) Simulated gain and directivity at θ = 0°, ϕ = 0° and (**b**) radiation efficiency as a function of the coating permittivity (1–mm thick coating thickness) at 406 MHz.

Several implanted antennas have been proposed in the literature for ISM bands and MICS [35,36,37]. A ceramic (alumina) antenna with a superstrate was proposed in [35]. Chip antennas made with low temperature co-fired ceramics (LTCC) are available on the market and are extensively used in wireless modules (ISM band, Bluetooth, GPS, WLAN, etc.). The high permittivity of these materials enables the antennas to be smaller so that they can be integrated into small circuits. In the present study, a miniature commercial LTCC ceramic antenna (0900AT43A0070, Johanson Technology, Camarillo, CA, USA) designed for operation in the air at 900 MHz is used to operate as an implant in the 402–433 MHz band. The antenna is 7.7 mm long, 2 mm wide, and 0.8 mm high. Simulations and experiments were conducted to study the feasibility of commercial ceramic antennas for devices implanted in MICS and the 433 MHz band. The small size of the antenna (0.0715*λ* in the body) means that it is electrically small compared with the wavelength, so neither the pattern diagram nor the directivity will be greatly affected, and it has an omni-directional radiation pattern. Due to the high permittivity surrounding the antenna, the effective electrical length of the monopole decreases and changes the antenna impedance. This drawback can be overcome with a matching network that has discrete components (inductors and capacitors) as the manufacturer proposes for air but tuned to the impedance of the implanted antenna. However, the main drawback is that the antenna radiation efficiency is reduced by the lossy tissue material close to the antenna. This reduction in radiation efficiency, in turn, reduces the antenna gain and from Equation (6), we determine that the *RCS_dif_* and the backscattering power are also lessened. Implanted medical devices must be covered by a biocompatible material to prevent contamination [38] and metallic oxidation, and the possibility of a short-circuit due to the high conductivity of the tissues [39]. The coating material acts as a superstrate and the thickness and the material have a considerable influence on the radiation efficiency [35]. Increasing the thickness of this coating material or the air gap between the cover and the antenna improves the radiation efficiency [39] but also increases the size of the implanted device. Therefore, there is a constraint between radiation efficiency and size. In the present study, we have used rubber silicone (*ε_r_* = 2.9, tan *δ* = 0.02) [40]. Figure 8 shows the reflection coefficient of the antenna connected to a short transmission line (width 1.85 mm) printed on 32 mil Roger RO4003C substrate (*ε_r_* = 3.55, tan *δ* = 0.0027) within the body with a 1 mm thick coating of silicone. The size of the ground plane was 10 mm × 10 mm. An image is shown in Figure 8a. This figure shows that the antenna is completely mismatched in the 400–433 MHz band. Therefore, a matching network is required. Assuming a well-matched design, the realized antenna gain must be close to the antenna gain that does not take into account the antenna mismatch. Figure 9 shows the gain, directivity, and radiation efficiency at 406 MHz as a function of the thickness of the silicone coating. The simulations were performed with the full-waveAnsys high-frequency structure simulator (HFSS) simulator including the model provided by the antenna manufacturer. We used a box of muscle tissue (*ε_r_* = 57, *σ* = 0.8 S/m) surrounding the antenna to simulate the effect of the body. The antenna directivity is not hardly affected by the thickness of the coating, but the radiation efficiency, and therefore the gain, increases with thickness. The efficiency fluctuates between 9.5% and 16%, which is considerably lower than in air (about 27%). Figure 10 shows the effect of the coating permittivity. This figure assumes the coating material to be 1 mm thick. The radiation efficiency increases slightly as the permittivity of the coating increases. However, it should be pointed out that coatings based on ceramic substrates with high permittivity are difficult to drill and cut.

**Figure 11:**
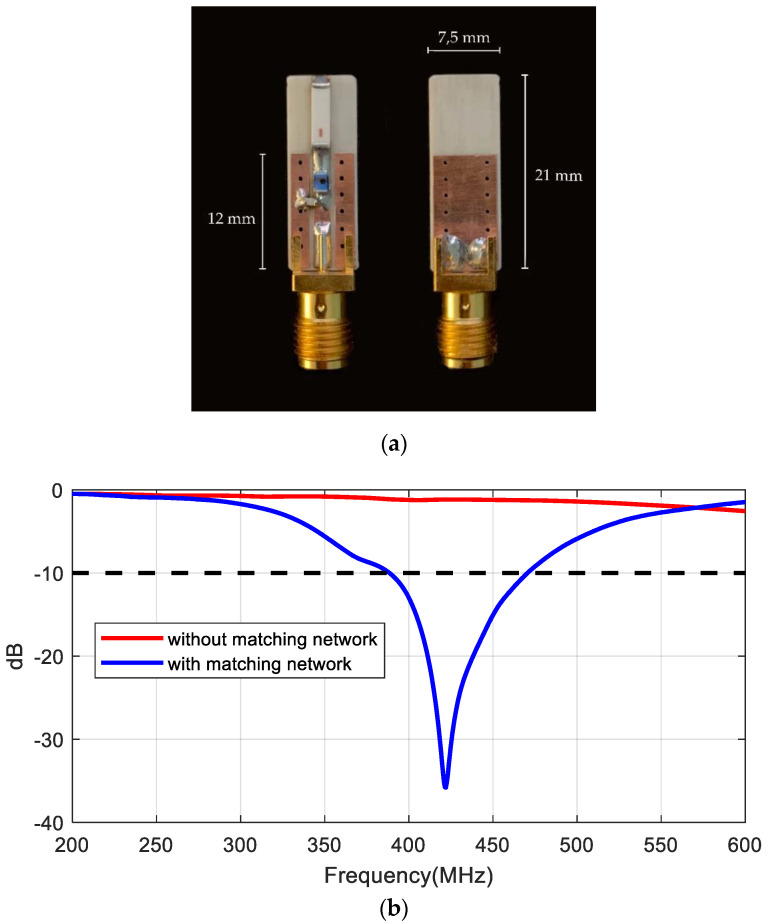
(**a**) Photography of the prototype manufactured for the antenna characterization, (**b**) measured reflection coefficient of the antenna in the phantom with and without the matching network.

In order to design the matching network with real materials and dimensions, a prototype with an SMA connector was manufactured (Figure 11a). The size of the board (7.5 mm × 21 mm) and ground plane (7.5 mm × 12 mm) together with the position of the discrete matching components is the same as in the implanted prototype (Figure 2). The impedance of the antenna was measured with a VNA connecting a thru line (zero Ohm SMD resistance), after which the reference plane was shifted to obtain the antenna impedance. Then, an LC matching network was designed using the Smith chart. Figure 11b shows the measured reflection coefficient of the antenna in the phantom material before and after the matching network. The frequency band can be tuned by modifying the components of the matching network. A 56 nH inductance in series and a 0.5 pF in parallel were used to match the antenna at 406 MHz and there was less than −10 dB between 387.28 MHz and 469.3 MHz (82.1 MHz bandwidth or 19.2%).

Table 1 compares the designed antenna with other printed implanted antennas proposed in the literature in the MICS band ([41,42,43,44]) using different miniaturization techniques. The miniature antenna proposed in the present study achieves a high gain and radiation efficiency, and a bandwidth that is moderate but enough to cover the MICS band. It is also available commercially, which simplifies the commercialization of future implanted devices.

### 2.5. Link Budget

In this section, a link budget will be presented to calculate the maximum depth and coverage area at which the implanted backscatter can be read as a function of the system parameters. The received power can be calculated using the Friis transmission equation with the following parameters: the transmission power (*P_Tx_*), the transmission antenna gain *G_Tx_* and receiver antenna gain *G_Rx_*, the wavelength *λ*, the differential RCS given by (6), the distance between the transmitter and the body (*d_Tx_*) and between the body and receiver (*d_Rx_*), the body attenuation *α*(dB/m), and the implant depth (*d*):

(8) $$P_{Rx}=\frac{P_{Tx}G_{Tx}}{4\pi d_{Tx}^{2}}{RCS}_{dif}10^{-0.1\alpha \cdot 2d}\frac{1}{4\pi d_{Rx}^{2}}\left(\frac{\lambda^{2}}{4\pi }\right)G_{Rx}$$

The parameters used in the simulations are listed in Table 2. The LoRa receiver sensitivity is a function of the noise figure of the receiver and the bandwidth *BW*. It is computed from the noise floor plus the required signal to noise ratio (*SNR*), which is a function of the Spreading Factor as shown in Table 3 [6,45]:

(9) S(dBm)=−174+10log(BW)+NF(dB)+SNR(dB)

One of the following values must be chosen as the bandwidth: 7.8, 10.4, 15.6, 20.8, 31.5, 41.7, 62.5, 125, 250, 500 kHz. The calculation of the sensitivity in Table 3 assumes a typical *BW* of 125 kHz. Thus, sensitivity decreases if lower bandwidth is used in accordance with (9) (12 dB for the lower bandwidth compared to 125 kHz). Table 3 also shows the time on air and the equivalent bit rate computed from the equations given in [6,45] considering 10 bytes of payload with a preamble of 6 bytes and a coding rate of CR = 4/5.

However, due to multipath propagation and reflections on the ground and objects, in indoor environments, the power decays with exponential factors that are not those given in the Friis model considered in (8). The received power or RSSI can be expressed using the following empirical model, which is an extension to the backscatter channel of the empirical propagation model for indoor environments [41,46]:

(10) $$P_{R}\left(d_{T_{x}},d_{R_{x}}\right)\left(dBm\right)=P_{R}\left(d_{0},d_{0}\right)\left(dBm\right)-10n_{1}\log\left(\frac{d_{T_{x}}}{d_{0}}\right)-10n_{2}\log\left(\frac{d_{R_{x}}}{d_{0}}\right)+X$$

In this model, the received power is modeled as an average term and a random term *X*, where *d*_0_ is a reference distance (e.g., the midpoint between transmitter and receiver *d*_0_ = (*d_Tx-Rx_*/2)) and *P_R_*(*d*_0_, *d*_0_) is the average received power at this reference distance. The exponential decay factors *n*_1_ and *n*_2_ are a function of the environment and the antenna height but for indoor environments, they have values of about 2.5–3 [47,48]. From the Friis equation, *P_R_*(*d*_0_, *d*_0_) is a function of transmitted power and the antenna gains. However, it is also a function of the antenna height and the diffraction. *P_R_*(*d*_0_, *d*_0_) can be found experimentally by regressing the measured data. Nevertheless, in this section, we perform simulations by considering the following expression derived from the Friis transmission Equation (8):

(11) $$\begin{array}{ll} P_{R}\left(d_{0},d_{0}\right)\left(dBm\right) & \\ & =P_{T}\left(dBm\right)+G_{T}\left(dB\right)+G_{R}\left(dB\right)-20\log\left(4\pi \right)+10\log\left(\frac{\lambda^{2}}{4\pi }\right)\\ & +{RCS}_{dif}\left(dB\right)-\alpha \left(dB/m\right)\cdot 2d-10n_{1}\log\left(d_{0}\right)-10n_{2}\log\left(d_{0}\right)\\ & -L_{obs}\left(dB\right) \end{array}$$

*L_obs_* is a term in dB that accounts for the attenuation for diffraction that depends on the antenna height [46]. The losses for ground diffraction can be neglected if the antenna height is higher than 0.6*R*_1_, where *R*_1_ is the radius of the first Fresnel zone [46]:

(12) $$R_{1}=\sqrt{\lambda \frac{d_{T_{x}}d_{R_{x}}}{d_{T_{x}}+d_{R_{x}}}}$$

For a distance between the transmitter and receiver (backscatter) of 2 m, and antennas of the same height, the minimum antenna height is 0.36 m at 406 MHz. Therefore, for a typical antenna height of around 1–2 m, we can expect that the effect of ground diffraction will be small.

The random variable *X* takes into account the attenuation due to multipath propagation. It can be regarded as a log-normally distributed random variable *X*(dB)~*N*(0,*σ*), where *σ* in dB is the standard deviation and experimental results have given values between 2 and 4 dB for a single path [48] (in our case we expect it to be double this because of the backscatter channel).

The coverage probability (*Prob*) is the probability that the average received power or RSSI, *P_R_*(dBm), will be higher than the receiver sensitivity *S*. For a log-normal distributed channel, it is given by [46]:

(13) $$Prob(P_{R}>S)=\frac{1}{2}-\frac{1}{2}erf\left(\frac{S-P_{R}}{\sqrt{2}\sigma }\right)$$

where *erf* is the Gauss error function. For *σ* = 8 dB and received power of 10 dB over the sensitivity (fading margin), the probability values are about 90%.

**Figure 12:**
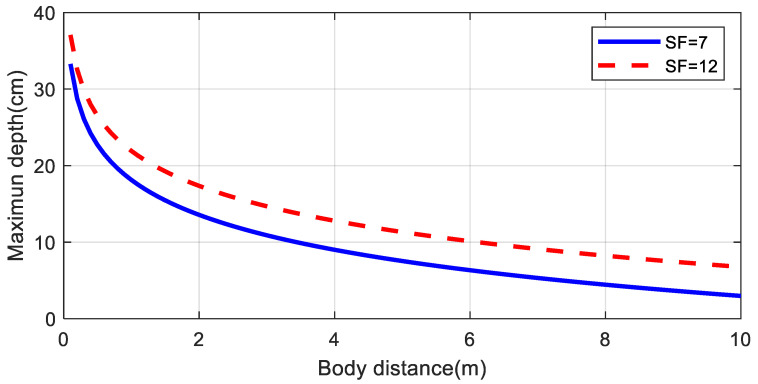
Simulated maximum depth for an implanted backscatter as a function of the distance to the body using the parameters of Table 1 for the monostatic case with a spreading factor *SF* = 7 and 12. Propagation model parameters: *n*_1_ = *n*_2_ = 2.5, Fade Margin = 10 dB.

**Figure 13:**
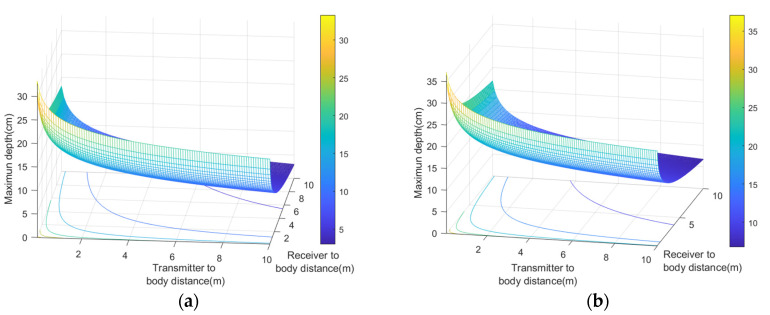
Simulated maximum depth for an implanted backscatter as a function of the distance of the transmitter and receiver from the body using the parameters of Table 2 for the bistatic case with a spreading factor *SF* = 7 (**a**) and *SF* = 12 (**b**). Propagation model parameters: *n*_1_ = *n*_2_ = 2.5, Fade Margin = 10 dB.

Two cases were analyzed: monostatic and bistatic. In the monostatic case, the LoRa transmitter and receiver gateway are close together (*d_Tx_* = *d_Rx_*). Figure 12 compares the maximum depth or distance inside the body that the backscatter can communicate for *SF* = 7 and *SF* = 12 with *BW* = 125 kHz. Figure 13 shows the maximum depth as a function of the transmitter to body distance and receiver to body distance in the bistatic case for *SF* = 7 and *SF* = 12 with *BW* = 125 kHz, respectively. In these simulations, the exponential decay factors (*n*_1_ and *n*_2_) were considered to be equal to 2.5. Depths can be greater than 10 cm at 4 m. In the bistatic case, depths can be greater if the transmitter is closer to the body than the receiver. These simulations have taken into account a fading margin due to multipath propagation of 10 dB. Results show the potential to read low rate data from implants using frequency-shifted LoRa backscatter. The number of packets per unit time increases if the spreading factor is low (e.g., *SF* = 7) as can be seen in Table 3 because the time on air is shorter than at high spreading factors. However, the sensitivity and the robustness of the system were better when spreading factors were high. In the bistatic case, to achieve better coverage, it is assumed that the transmitter will be located near the patient (e.g., close to the bed) and the receiver somewhere else in the room.

**Figure 14:**
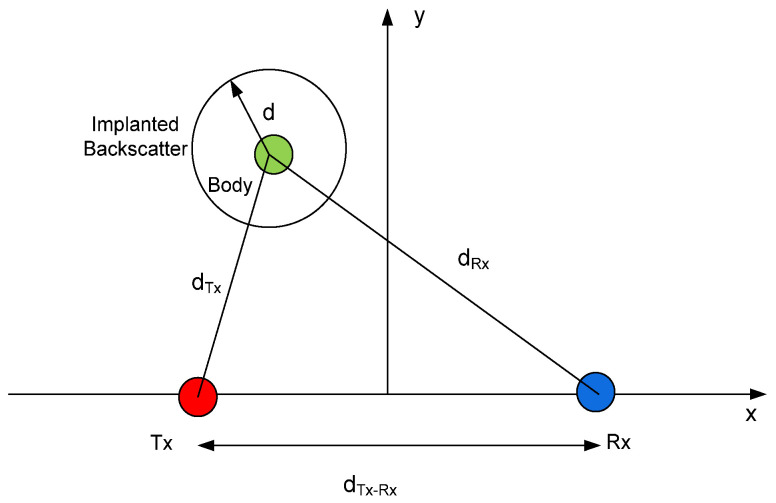
Scheme used in the RSSI (Received Signal Strength Indicator) simulations.

**Figure 15:**
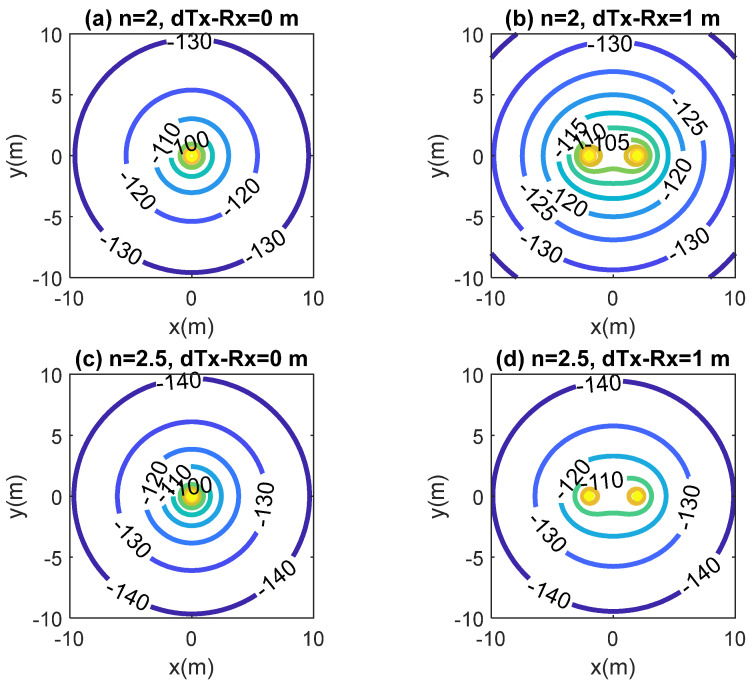
Simulated RSSI as a function of the location of the implanted backscatter assuming a depth of 5 cm inside the body for the monostatic case (*d_Tx-Rx_* = 0 m) and bistatic case (*d_Tx-Rx_* = 1 m). Path loss exponential factor *n*_1_ = *n*_2_ = 2 and *n*_1_ = *n*_2_ = 2.5.

**Figure 16:**
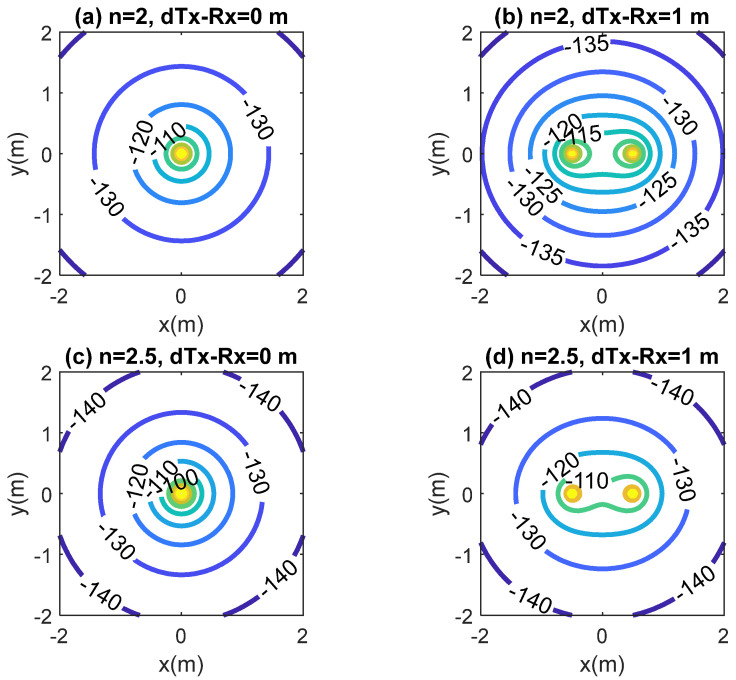
Simulated RSSI as a function of the location of the implanted backscatter assuming a depth of 10 cm inside the body for the monostatic case (*d_Tx-Rx_* = 0 m) and bistatic case (*d_Tx-Rx_* = 1 m). Path loss exponential factor *n*_1_ = *n*_2_ = 2 and *n*_1_ = *n*_2_ = 2.5.

A simplified scenario shown in Figure 14 consists of the transmitter and the receiver and the implanted backscatter on the same plane. All these elements are used to estimate the coverage from the propagation model proposed. In this scenario, it is assumed that the transceivers are fixed and the backscatter can move around different positions. Figure 15 and Figure 16 show the simulated coverage results for backscatter at a depth (d) of 5 cm and 10 cm, respectively. They depict the average received power computed with (10)–(11) considering the ideal free space exponential factors *n*_1_ = *n*_2_ = 2 and *n*_1_ = *n*_2_ = 2.5 as a function of the position of the backscatter. The monostatic and biostatic case (with a distance between the transmitter and receiver *d_Tx-Rx_* = 1 m) are shown. For devices implanted on the surface or at small depths (<5 cm), the read range area can be about 4 m × 4 m, whereas for deep implanted devices (10 cm), a read range area of about 1.8 m × 1.8 m can be covered.

## 3. Results

**Figure 17:**
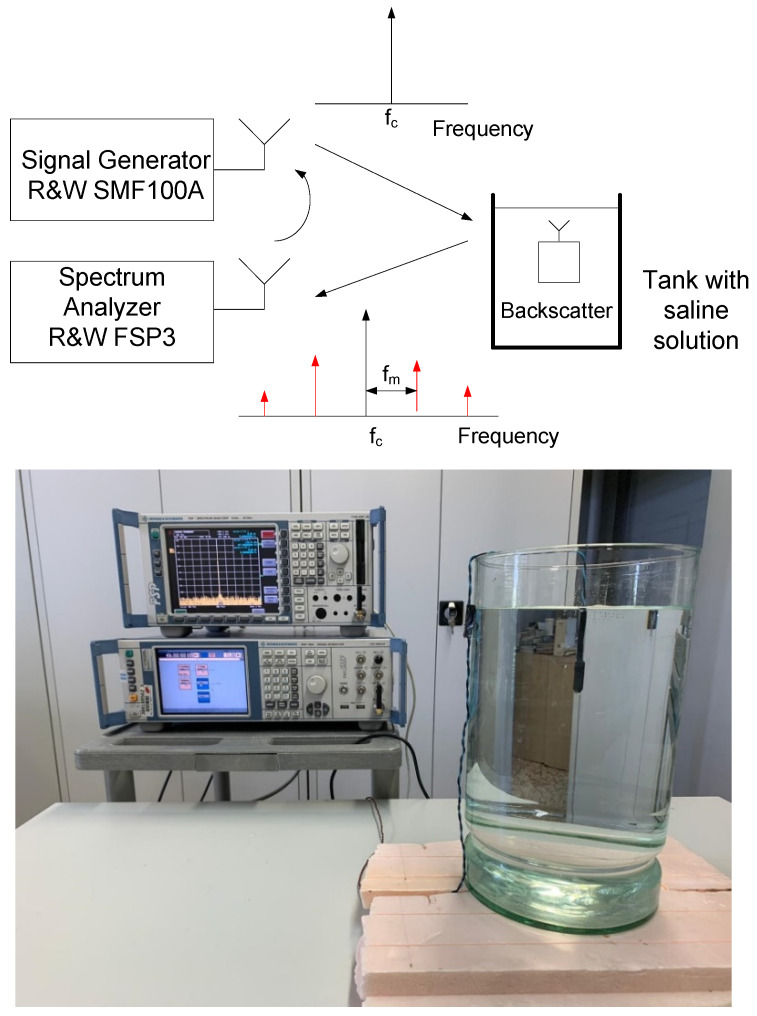
Block diagram of the setup used for the characterization of the backscatter and photograph (bottom).

**Figure 18:**
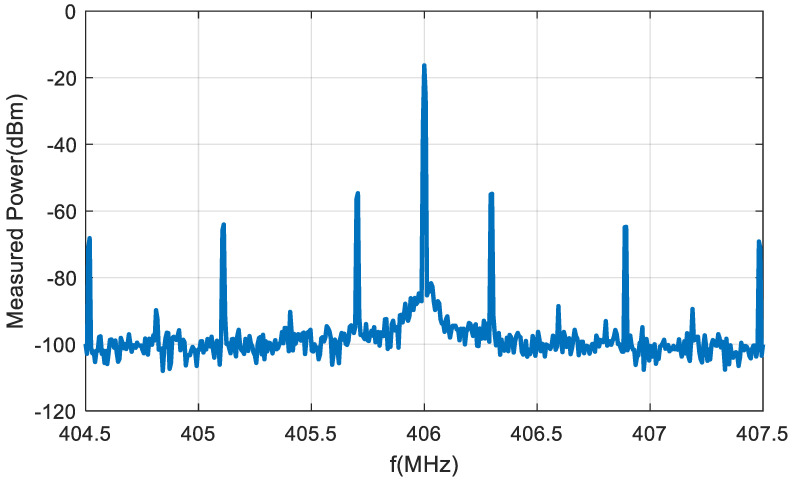
Spectrum of the receiver signal when the backscatter is illuminated at 406 MHz using the setup in Figure 17.

**Figure 19:**
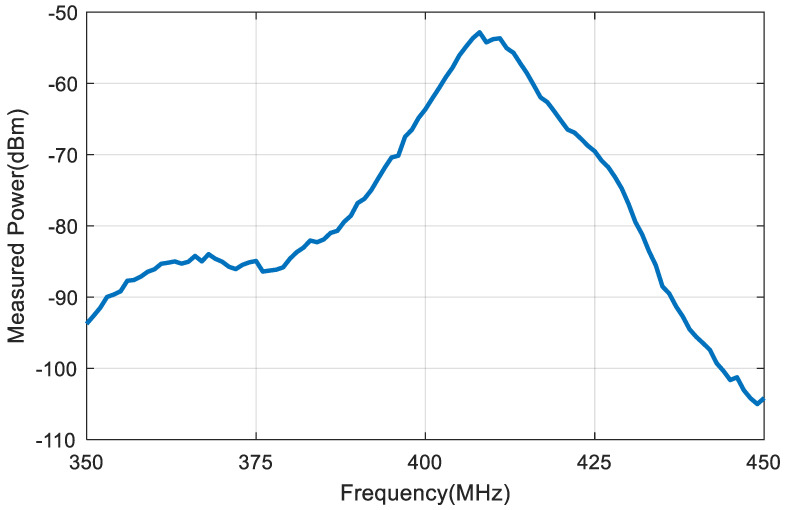
First harmonic (*f_c_* + *f_m_*) measured power as a function of the frequency with the backscatter immersed in the vessel described in Figure 17.

A backscatter prototype has been designed as shown in the scheme in Figure 1. The prototype includes an antenna with a matching network, an RF switch (ADG902) used as a modulator, and a low-power oscillator (LTC6907). The system is controlled by a microcontroller (ATTiny85) [49] that can generate test messages. The backscatter is covered with 1 mm of heat shrink silicone (approximately 1 mm thick). This proof of concept prototype is externally biased with a battery pack connected by two wires that are also used to support the device. A photograph is shown in Figure 17. In order to check the correct modulation of the backscatter inside the body, the backscatter was inserted into a 20 cm diameter cylindrical vessel (see Figure 17). The vessel was filled with a mixture of saline solution with sugar described in the previous section. A signal generator was connected to a transmitting antenna and a spectrum analyzer was used as a receiver connected to a receiving antenna. Two whip monopoles tuned to 406 MHz were used in the generator and the spectrum analyzer. The distance from the Tx and RX to the vessel was 1 m. The frequency was swept and the backscattered power was received. Figure 18 shows the spectrum in which the sidebands at the modulating backscatter frequency at 406 MHz can be seen. The central peak at this carrier frequency was due to the coupling between the transmitter and receiver. The sideband peaks are spaced from the carrier at the same frequency at which the ADG902 is switching (300 kHz in this experiment). Figure 19 shows the measurement of the received power at the sideband as a function of the generator frequency. The maximum received power was around the 408 MHz band. It is observed that the backscatter covers the desired frequency band, which shows that the antenna was correctly tuned within the phantom.

**Figure 20:**
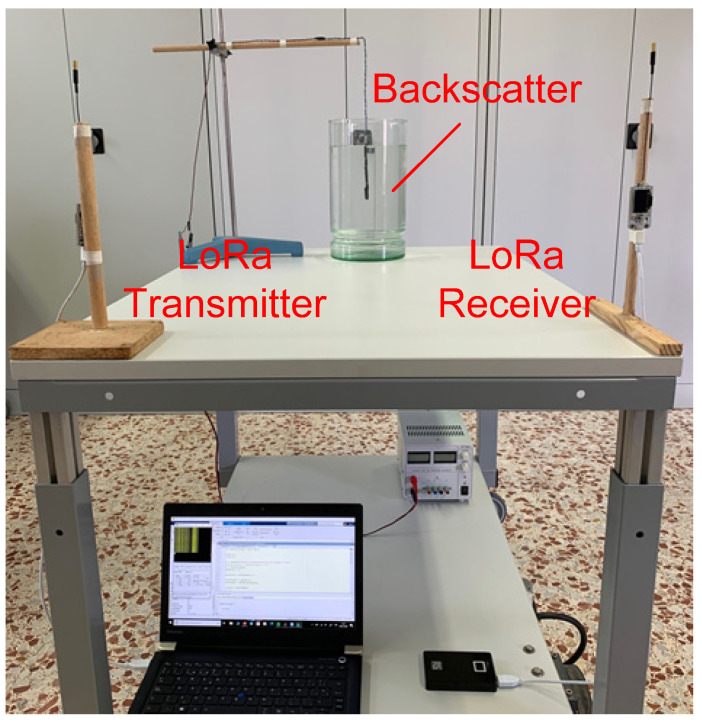
Photograph of the experimental setup in the laboratory.

To test the reliability of the system, some experimental results were achieved using a LoRa transmitter (ESP32 TGNO with Semtech SX1278 transceiver) and another identical LoRa module used as a receiver. The central frequency of the transmitter was 406 MHz and the oscillation frequency was 300 kHz. The receiver was tuned to 406.3 MHz. The LoRa was configured to transmit and receive packets with *SF* = 12 and *BW* = 125 kHz for better sensitivity. Figure 20 shows an image of the setup used in the experiments.

**Figure 21:**
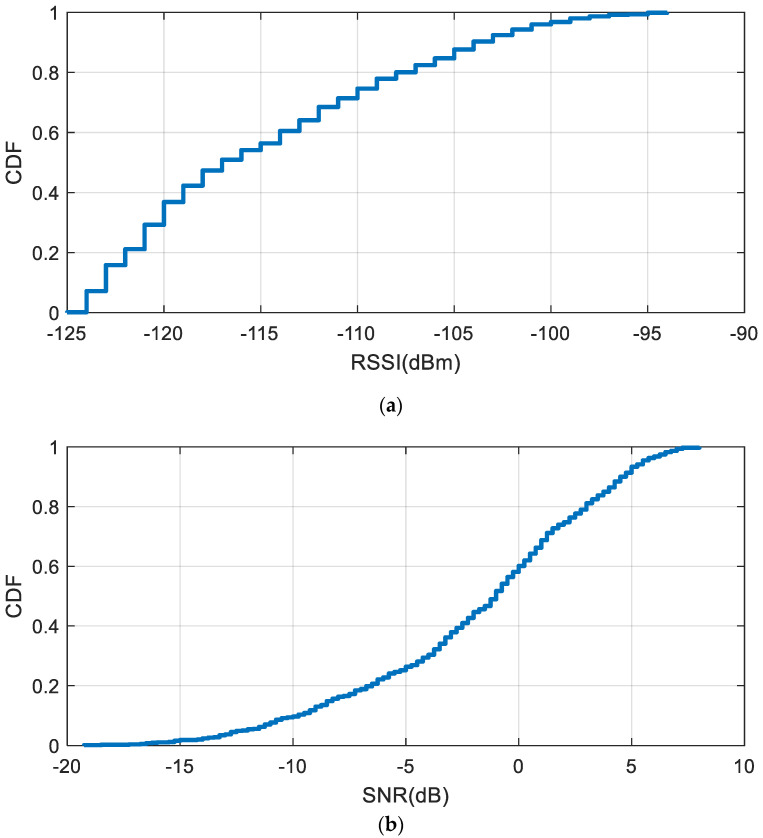
Complementary distribution function of the measured RSSI (**a**) and SNR (signal to noise ratio) (**b**) with the backscatter immersed in the vessel for a transmitter located 80 cm from the vessel.

Figure 21 shows the complementary distribution function (CDF) plot of the received signal strength indicator (RSSI) and the SNR in the shifted channel measured by the LoRa transceiver. In order to obtain the data, the receiver was tuned to the backscattered channel and was located at random points (uniformly distributed) in the laboratory within a coverage area of 4 × 4m. The distance from the LoRa transmitter to the vessel was 80 cm at a height of 1 m from the ground. The backscatter was immersed in the center of a 20 cm diameter vessel with the phantom solution. We can observe that the RSSI is typically higher than −123 dBm (less than 10% of the locations receive levels below −123 dBm and SNR below −15 dB). Therefore, most locations were above the sensitivity of the receiver, and the packets were successfully backscattered.

**Figure 22:**
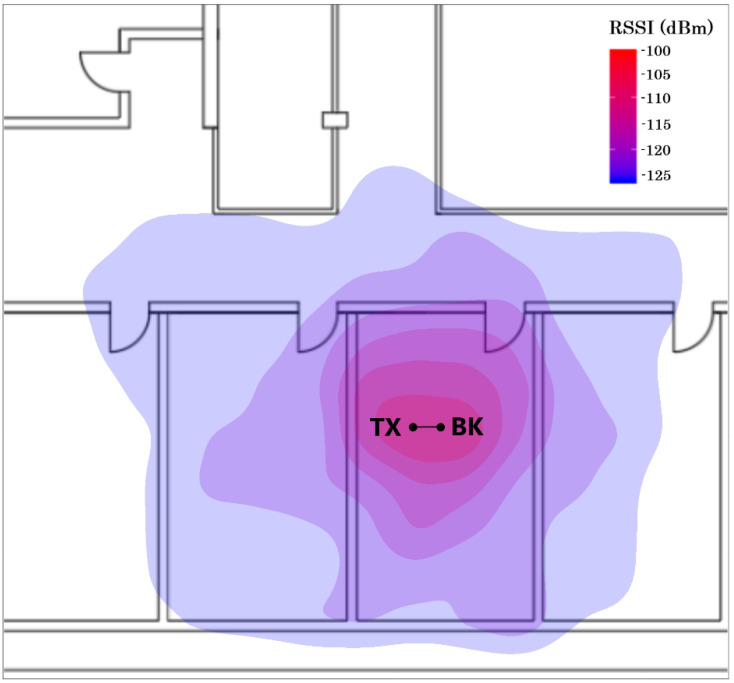
Measured RSSI heat map for the transmitter located 0.8 m from the vessel with the backscatter immersed (the points marked as BK and TX indicate the location of the backscatter and the transmitter, respectively).

In order to show the coverage area, Figure 22 shows the heat maps of the measured RSSI for the transmitter located 80 cm from the vessel with the immersed backscatter prototype centered and immersed at a depth of 10 cm The backscatter prototype and the transmitter are fixed whereas the receiver is moved along different locations in the room (laboratory). Under these circumstances, data can be received within the laboratory (area 5 m × 10 m) and through walls provided the receiver is located in neighboring rooms on the same floor. This result is in accordance with Figure 13b which shows that devices implanted at a 10 cm depth can be detected up to 10 m from the receiver when the transmitter is closed (80 cm in this case).

**Figure 23:**
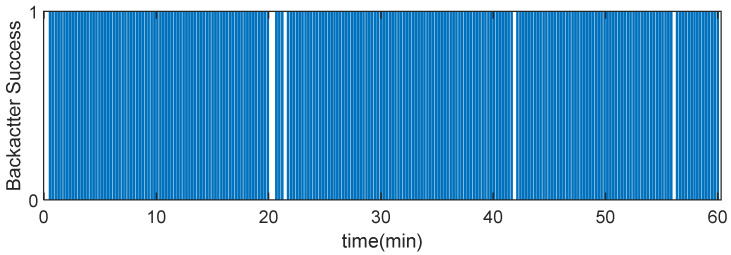
Backscattered frames successfully received as a function of the time with the backscatter immersed in the center of the vessel with a phantom liquid for a transmitter located 0.8 m from the vessel.

To emulate the functionality of an implanted sensor, the measurement of the temperature with an external sensor is transmitted using the encoding procedure described above. Figure 23 shows the received data obtained with frame transmission over 60 min. The transmitter was located 80 cm from the vessel surface with the backscatter immersed in the phantom liquid at the center. The receiver was located at a distance of 1.5 m. In this test, the better range *SF* configuration [3] was set up (*SF* = 12), keeping the bandwidth (*BW)* to 125 kHz to not increase drastically the time on air of the packet. To test the backscatter, a straightforward protocol (described in Section 2.2) to send a byte in a 10 bits frame (start bit plus 8 data bits and end of frame bit) was implemented. The average number of frames lost was 4%. These results pave the way for long-range wireless implanted devices based on backscattering communication in indoor environments.

## 4. Discussion

The use of a frequency band (406–433 MHz) allows to increase the coverage because the propagation losses are lower than at 868/915 MHz or 2.45 GHz ISM band (free space attenuation decays with the square of the frequency) and besides, the walls [50] and indoor propagation [48] introduce lower attenuation at this frequency band. This helps to counter the fading effect caused by multipath propagation in indoor environments which, in backscattering systems, affects the up and downlinks [51,52,53]. The proposed system can easily use receiver diversity with multiple receivers to sniff shifted channels and prevent selective multipath fading. Channel hopping is not expected to provide significant improvements in the 406 MICS and 433 MHz ISM bands due to the small spacing between channels (less than the channel coherence bandwidth) so the channels are correlated [47,48]. However, to prevent interference or collision with another system, a listen-before-talk procedure (LBT) can easily be implemented in the LoRa transmitter so that the available channels can be listened to before the packets are sent. The parameters of the channel (frequency, *SF*, *BW*) can be transmitted to the receivers using a conventional LoRa link. This is different from the 2.4 GHz band, where channel hopping can be used to counter the effects of multipath propagation (e.g., in Bluetooth) [48].

This section compares the technology presented with other existing wireless technologies for implanted applications. Table 4 summarizes some examples of wireless technologies and compares such parameters as the carrier frequency, data rate, implant depth, or read range. These technologies can be classified according to whether are battery-less or not. Each technology has advantages and drawbacks depending on the requirements of the final application. Due to the higher losses of the body, Wireless Power Transfer (WPT) based on inductive links at low-frequency (LF) [54] and high-frequency (HF) [55,56] is the method preferred to power battery-less devices. Data are transferred by load modulation (Load Shift Keying-LSK) or by another wireless link at higher frequencies [57]. Depth and power depend on the size of the coils used [55], and the communication (read range) can be performed when the coils are close to the body. Another drawback is that it requires specialized readers and ASICs. In [58], the authors propose a method based on three coils to improve the depth and reading range using smartphones with NFC and commercial NFC IC with energy-harvesting capabilities.

When the carrier frequency increases, the communication uses far-field and the attenuation increases very quickly with the frequency. Passive UHF RFID systems for identification and sensor communication at small implanted depths (5–15 mm subcutaneous) have been proposed [59,60]. The communication is by backscattering and a commercial UHF reader can be used. The WPT link is limited by the sensitivity of the UHF RFID IC [60] (about −20 dBm for modern ICs such as Impinj Monza R6 and −10 dBm for AMS-SL900A with sensing capabilities). The reader transmitter power is limited by exposure regulations (SAR) [60] so when body losses are higher the read range is limited to 0.6–1.6 m depending of the depth of the implant (5 to 15 mm) [59].

Battery implanted devices are used when high performance and high data rate transfer are required. Commercial active implantable transceivers are available on the market. For example, the Microsemi ZL70103 transceiver [61] operates in the MICS band and the 433 MHz ISM band at a raw data rate of up to 400 kbps and with wake-up at 3.45 GHz. Its Rx sensitivity is −102 dBm at 200 kbps (with a packet error rate of 10%) and the Tx/Rx average current is 5 mA. Several implanted wireless devices have been proposed for endoscopy [62,63,64,65,66]. For this application to download video data, a high data rate is needed; therefore, active transceivers are used. However, the battery lifetime is limited to a few hours, which is enough for the exploration period required. Data rates from 1 Mbps [66] to 80 Mbps [65] have been achieved using different modulations such as BPSK [65], FSK [62], QPSK [63,64], or OFDM [66], at frequency bands ranging from 20 MHz to 925 MHz transmitting power between 1 mW and 6 mW. The price to pay is high power consumption and, therefore, the limited lifetime. It is not the best option for long term monitoring even if the data rate required is small.

Zero power backscatter gives better power consumption because it does not transmit RF power, which extends the life of the battery although the link attenuation is double that of active transceivers due to the forward and backscattered channel. The proposed backscatter can be used with other modulations such as ASK (OOK) or FSK and the bitrate achieved (a few MHz) is high enough to transfer implanted video camera data. In this case, a CW tone illuminates the backscatter from the transmitter and the backscatter responds by modulating the carrier. A backscatter similar to that described in this study was used in [9] to achieve 30 Mbps working at 915 MHz at a depth of 6 cm using a loop antenna. In another study [67], an FSK with a backscatter achieved 1–5 Mbps at 600 MHz and a depth of up to 10 cm. In both cases, the reader antennas were located close to the phantom and the reader system was implemented using software-defined radio (SDR). The LoRa backscatter technique proposed in this study can achieve greater depths and read ranges outside the body because of the higher sensitivity of the LoRa modulation, but the main limitation is the low data rate. Another important point is that the proposed system does not require specialized readers because commercial low-cost LoRa transceivers can be used.

## 5. Conclusions

This paper shows the feasibility of communication based on receiving backscatter LoRa packets at a shifted channel for implanted devices. A proof of concept prototype has been developed. The prototype consists of an RF switch that presents two high reflective coefficients to an antenna controlled by a low-frequency modulating oscillator. A commercial ceramic antenna is tuned using a matching network at the MICS and ISM 433 MHz bands, which provides high miniaturization and high radiation efficiency. The backscatter can be interfaced with a low power microprocessor to send low rate data from sensors for purposes of telemetry. Although the backscatter can be used with other modulations such as ASK and FSK, the proposed method uses the reflection of LoRa packets to exploit the high sensitivity and robustness of LoRa modulation; therefore, there is no need to integrate high power-consuming FPGAs or other ICs. A complete link budget has been presented which shows the potentiality of the communication technique. The method proposed achieves great depths at distances of up to several meters from the body so that only one transmitter base station is enough to cover a typical room. Conventional LoRa transceivers are compatible with the proposed backscatter system, which can be used for long-range monitoring in indoor environments for future implanted and wearable devices.

## Figures and Tables

**Table 1:** Comparison with other antennas in the Medical Implant Communication Service (MICS) band reported in the literature.

Reference	Volume (mm^3^)	Antenna Type	Gain (dB)	Efficiency (%)	*BW* at −10 dB (MHz)
F-J. Huang et al. [41]	245	PIFA with superstrate	−7	39	115
A. Kiourti et al. [42]	32.7	Meander patches	−45	0.81	40
W.C. Liu [43]	190	Spiral patch	−26	0.61	50
D. Nikolayev et al. [44]	705	Alumina capsule, λ/2 SIR	−22	0.4	16
This study	28 (236 ^1^)	Ceramic SMD	−9.5	10.8	82.1

^1^ Including a coating of 1 mm and a matching network and ground plane.

**Table 2:** Parameters used in link budget.

Parameter	Value	Unit
Transmission power *P_Tx_*	20	dBm
Transmission antenna gain *G_Tx_*	0	dB
Receiver antenna gain *G_Rx_*	0	dB
Tag antenna gain *G_a_*	−10	dB
Carrier frequency	406	MHz
Attenuation per unit length *α*	165	dB/m
Exponential decay factors *n*_1_ and *n*_2_	2.5	
|Γ_ON_-Γ_OFF_|^2^	2.8 ^1^	
Modulation factor *m*	1/π^2^	
Noise figure of the receiver *NF*	6	dB
Bandwidth *BW*	125	kHz

^1^ From measurements shown in Figure 4.

**Table 3:** Parameters as a function of the Spreading Factor.

Spreading Factor *SF*	Chips/Symbol ^1^	*SNR* (Signal to Noise Ratio) (dB)	Time on Air of a 10-byte Packet ^2^ (ms)	Bit rate (bps)	Sensitivity *S* (dBm) for *BW* = 125 kHz ^3^
7	128	−7.5	56	5470	−124.5
8	256	−10	103	3125	-127.0
9	512	−12.5	205	1758	−129.5
10	1024	−15	371	977	−132.5
11	2048	−17.5	741	537	−134.5
12	4096	−20	1483	293	−137.0

^1^ Chips/Symbol = 2^SF^. ^2^ with a preamble of 6 symbols and CR = 4/5. ^3^ *S* can be reduced 12 dB for *BW* = 7.8 kHz.

**Table 4:** Comparison with other communication technologies for implantable medical devices.

Ref.	Technology /Power	Carrier Frequency	Modulation	Implanted Depth	Link Range Outside the Body	Bitrate (bps)	Base Station Receiver Sensitivity (dBm)
[54]	Inductive /RF	700 kHz	CW (WPT)	20 mm	BS ^1^	-	-
[56]	Inductive /RF	13.56 MHz	LSK, ASK	15 mm	BS ^1^	2 Mbps	NA
[57]	Inductive /RF	10 MHz	PPSK, OOK	8 mm	BS ^1^	1.35 Mbps	NA
[58]	NFC /RF	13.56 MHz	LSK, ASK	15–20 mm	3 cm	26.4/848 kbps ^2^	Depends on reader
[59,60]	UHF RFID /RF	868/915 MHz		5–15 mm	0.6–1 m	40 kbps	−60/−70
[61]	Active /Battery	406/433 MHz 2.45 GHz Wakeup	OOK	NA	10–20 cm	400 kbps	−91
[62,63,64,65,66]	Endoscopy /Battery	20 to 925 MHz	BPSK, QPKS, FSK, OFDM	10–20 cm	BS ^1^		−85
[67]	Backscattering /Battery	600 MHz	FSK	10 cm	BS ^1^	1–5	−87
[9]	Backscattering /Battery	915 MHz	ASK	6 cm	BS ^1^	30	−90
This work	LoRa backscattering /Battery	406–433 MHz	LoRa	10–20 cm	<4 m	Few bps	−137

^1^ Receiver antenna on body surface. ^2^ The data rate depends on standard ISO15693 (26.4 kbps) ISO1443 (up to 848 kbps).

## References

[1] Adelantado F., Vilajosana X., Tuset-Peiro P., Martinez B., Melia-Segui J., Watteyne T. (2017). Understanding the Limits of LoRaWAN. IEEE Commun. Mag..

[2] De Carvalho Silva J., Rodrigues J.J.P.C., Alberti A.M., Solic P., Aquino A.L.L. LoRaWAN—A low power WAN protocol for Internet of Things: A review and opportunities. Proceedings of the 2nd International Multidisciplinary Conference on Computer and Energy Science (SpliTech).

[3] Bankov D., Khorov E., Lyakhov A. On the Limits of LoRaWAN Channel Access. Proceedings of the 2016 International Conference on Engineering and Telecommunication (EnT).

[4] Casals L., Mir B., Vidal R., Gomez C. (2017). Modeling the Energy Performance of LoRaWAN. Sensors.

[5] Vangelista L. (2017). Frequency Shift Chirp Modulation: The LoRa Modulation. IEEE Signal Process. Lett..

[6] Semtech SX1276/77/78/79-137 MHz to 1020 MHz Low Power Long Range Transceiver. https://semtech.my.salesforce.com/sfc/p/E0000000JelG/a/2R0000001OKs/Bs97dmPXeatnbdoJNVMIDaKDlQz8q1N_gxDcgqi7g2o.

[7] Wixted A.J., Kinnaird P., Larijani H., Tait A., Ahmadinia A., Strachan N. Evaluation of LoRa and LoRaWAN for Wireless Sensor Networks. Proceedings of the 2016 IEEE SENSORS.

[8] Thomas S.J., Besnoff J.S., Reynolds M.S. Modulated Backscatter for Ultra-Low Power Uplinks from Wearable and Implantable Devices. Proceedings of the 2012 ACM Workshop on Privacy in the Electronic Society—WPES’ 12.

[9] Besnoff J.S., Reynolds M.S. Near Field Modulated Backscatter for in Vivo Biotelemetry. Proceedings of the 2012 IEEE International Conference on RFID (RFID).

[10] Vasisht D., Zhang G., Abari O., Lu H.-M., Flanz J., Katabi D. In-Body Backscatter Communication and Localization. Proceedings of the 2018 Conference of the ACM Special Interest Group on Data Communication.

[11] Khaleghi A., Balasingham I. Wireless Capsule Endoscopy Using Backscatter Communication. Proceedings of the 13th European Conference on Antennas and Propagation (EuCAP).

[12] Iyer V., Talla V., Kellogg B., Gollakota S., Smith J. Inter-Technology Backscatter. Proceedings of the 2016 Conference on ACM SIGCOMM.

[13] Liu W., Huang K., Zhou X., Durrani S. (2019). Next Generation Backscatter Communication: Systems, Techniques, and Applications. EURASIP J. Wirel. Commun. Netw..

[14] Van Huynh N., Hoang D.T., Lu X., Niyato D., Wang P., Kim D.I. (2018). Ambient Backscatter Communications: A Contemporary Survey. IEEE Commun. Surv. Tutor..

[15] Zhao J., Gong W., Liu J. X-tandem: Towards Multi-Hop Backscatter Communication with Commodity Wifi. Proceedings of the 24th Annual International Conference on Mobile Computing and Networking.

[16] Varshney A., Harms O., Pérez-Penichet C., Rohner C., Hermans F., Voigt T. Lorea: A Backscatter Architecture That Achieves a Long Communication Range. Proceedings of the 15th ACM Conference on Embedded Network Sensor Systems.

[17] Zhang P., Rostami M., Hu P., Ganesan D. Enabling Practical Backscatter Communication for On-body Sensors. Proceedings of the 2016 conference on ACM SIGCOMM.

[18] Peng Y., Shangguan L., Hu Y., Qian Y., Lin X., Chen X., Fang D., Jamieson K. PLoRa: A Passive Long-Range Data Network from Ambient Lora Transmissions. Proceedings of the 2018 Conference of the ACM Special Interest Group on Data Communication.

[19] Talla V., Hessar M., Kellogg B., Najafi A., Smith J.R., Gollakota S. (2017). LoRa Backscatter: Enabling the Vision of Ubiquitous Connectivity. Proc. ACM Interact. Mob. Wearable Ubiquitous Technol..

[20] Islam M.N., Yuce M.R. (2016). Review of Medical Implant Communication System (MICS) Band and Network. ICT Express.

[21] Green R.B. (1963). The General Theory of Antenna Scattering. Ph.D. Thesis.

[22] Collin R.E., Zucker F.J. (1969). The Receiving Antenna. Antenna Theory.

[23] Nikitin P., Rao K., Lam S., Pillai V., Martinez R., Heinrich H. (2005). Power Reflection Coefficient Analysis for Complex Impedances in RFID Tag Design. IEEE Trans. Microw. Theory Tech..

[24] ADG901/ADG902 Datasheet. https://www.analog.com/media/en/technical-documentation/data-sheets/ADG901_902.pdf.

[25] LTC6907-Micropower, 40 kHz to 4 MHz Resistor Set Oscillator in SOT-23. https://www.analog.com/media/en/technical-documentation/data-sheets/6907fa.pdf.

[26] (2015). 868 and 915MHz Dual (Wideband) ISM Band SMD Chip Antenna P/N 0900AT43A0070. https://www.johansontechnology.com/datasheets/0900AT43A0070/0900AT43A0070.pdf.

[27] Nikitin P.V., Rao K.V.S. Antennas and Propagation in UHF RFID Systems. Proceedings of the 2008 IEEE International Conference on RFID.

[28] Gabriel S., Lau R.W., Gabriel C. (1996). The Dielectric Properties of Biological Tissues: II. Measurements in the Frequency Range 10 Hz to 20 GHz. Phys. Med. Biol..

[29] Castello-Palacios S., Garcia-Pardo C., Alloza-Pascual M., Fornes-Leal A., Cardona N., Valles-Lluch A. (2019). Gel Phantoms for Body Microwave Propagation in the (2 to 26.5) GHz Frequency Band. IEEE Trans. Antennas Propag..

[30] Chou C.-K., Chen G.-W., Guy A.W., Luk K.H. (1984). Formulas for Preparing Phantom Muscle Tissue at Various Radiofrequencies. Bioelectromagnetics.

[31] Castello-Palacios S., Valles-Lluch A., Garcia-Pardo C., Fornes-Leal A., Cardona N. Formulas for Easy-to-Prepare Tailored Phantoms at 2.4 GHz ISM Band. Proceedings of the 11th International Symposium on Medical Information and Communication Technology (ISMICT).

[32] Peterson D.M., Turner W., Pham K., Yu H., Bashirullah R., Euliano N., Fitzsimmons J.R. A Tissue Equivalent Phantom of the Human Torso for in vivo Biocompatible Communications. Proceedings of the 26th Southern Biomedical Engineering Conference SBEC 2010.

[33] Burdette E., Cain F., Seals J. (1980). In Vivo Probe Measurement Technique for Determining Dielectric Properties at VHF through Microwave Frequencies. IEEE Trans. Microw. Theory Tech..

[34] Ayappa K., Davis H., Crapiste G., Davis E., Gordon J. (1991). Microwave Heating: An Evaluation of Power Formulations. Chem. Eng. Sci..

[35] Soontornpipit P., Furse C.M., Chung Y.C. (2004). Design of Implantable Microstrip Antenna for Communication with Medical Implants. IEEE Trans. Microw. Theory Tech..

[36] Murphy O.H., McLeod C.N., Navaratnarajah M., Yacoub M., Toumazou C. (2011). A Pseudo-Normal-Mode Helical Antenna for Use with Deeply Implanted Wireless Sensors. IEEE Trans. Antennas Propag..

[37] Kiourti A., Nikita K.S. (2012). A Review of Implantable Patch Antennas for Biomedical Telemetry: Challenges and Solutions [Wireless Corner]. IEEE Antennas Propag. Mag..

[38] Donaldson P.E.K., Sayer E. (1981). A Technology for Implantable Hermetic Packages. Part 2: An Implementation. Med. Biol. Eng. Comput..

[39] Merli F., Fuchs B., Mosig J.R., Skrivervik A.K. (2010). The Effect of Insulating Layers on the Performance of Implanted Antennas. IEEE Trans. Antennas Propag..

[40] Teo A.J., Mishra A., Park I., Kim Y.-J., Park W.-T., Yoon Y.-J. (2016). Polymeric Biomaterials for Medical Implants and Devices. ACS Biomater. Sci. Eng..

[41] Huang F.-J., Lee C.-M., Chang C.-L., Chen L.-K., Yo T.-C., Lai S.-C. (2011). Rectenna Application of Miniaturized Implantable Antenna Design for Triple-Band Biotelemetry Communication. IEEE Trans. Antennas Propag..

[42] Kiourti A., Christopoulou M., Nikita K.S. Performance of a Novel Miniature Antenna Implanted in the Human Head for Wireless Biotelemetry. Proceedings of the 2011 IEEE International Symposium on Antennas and Propagation (APSURSI).

[43] Liu W., Yeh F., Ghavami M. (2008). Miniaturized Implantable Broadband Antenna for Biotelemetry Communication. Microw. Opt. Technol. Lett..

[44] Nikolayev D., Zhadobov M., Le Coq L., Karban P., Sauleau R. (2017). Robust Ultraminiature Capsule Antenna for Ingestible and Implantable Applications. IEEE Trans. Antennas Propag..

[45] (2013). Semtech SX1272/3/6/7/8: LoRa Modem Designers Guide AN1200.13, Revision 1 Edition. https://www.rs-online.com/designspark/rel-assets/ds-assets/uploads/knowledge-items/application-notes-for-the-internet-of-things/LoRaDesignGuide.pdf.

[46] Goldsmith A. (2005). Wireless Communications.

[47] Lazaro A., Girbau D., Salinas D. (2009). Radio Link Budgets for UHF RFID on Multipath Environments. IEEE Trans. Antennas Propag..

[48] Tuset-Peiro P., Anglès-Vazquez A., Vicario J.L., Vilajosana X. (2013). On the Suitability of the 433 MHz Band for M2M Low-Power Wireless Communications: Propagation Aspects. Trans. Emerg. Telecommun. Technol..

[49] Atmel 8-bit AVR Microcontroller with 2/4/8K Bytes In-System Programmable Flash. ATtiny 25/V/ATtiny45/V/ATtiny85/V, Rev. 2586Q-AVR-08/2013. https://ww1.microchip.com/downloads/en/DeviceDoc/Atmel-2586-AVR-8-bit-Microcontroller-ATtiny25-ATtiny45-ATtiny85_Datasheet.pdf.

[50] Ali-Rantala P., Ukkonen L., Sydanheimo L., Keskilammi M., Kivikoski M. Different Kinds of Walls and Their Effect on the Attenuation of Radiowaves Indoors. Proceedings of the IEEE Antennas and Propagation Society International Symposium. Digest. Held in Conjunction with: USNC/CNC/URSI North American Radio Sci. Meeting (Cat. No.03CH37450).

[51] Lorenzo-Navarro J., Lazaro A., Girbau D., Villarino R., Gil E. (2016). Analysis of on-Body Transponders Based on Frequency Selective Surfaces. Prog. Electromagn. Res..

[52] Lorenzo J., Lazaro A., Villarino R., Girbau D. (2017). Diversity Study of a Frequency Selective Surface Transponder for Wearable Applications. IEEE Trans. Antennas Propag..

[53] Milici S., Lazaro A., Lorenzo J., Villarino R., Girbau D. Wearable Sensors Based on Modulated Frequency Selective Surfaces. Proceedings of the 47th European Microwave Conference (EuMC).

[54] Ramrakhyani A.K., Mirabbasi S., Chiao M. (2010). Design and Optimization of Resonance-Based Efficient Wireless Power Delivery Systems for Biomedical Implants. IEEE Trans. Biomed. Circuits Syst..

[55] Jiang D., Cirmirakis D., Schormans M., Perkins T.A., Donaldson N., Demosthenous A. (2016). An Integrated Passive Phase-Shift Keying Modulator for Biomedical Implants with Power Telemetry Over a Single Inductive Link. IEEE Trans. Biomed. Circuits Syst..

[56] Lin Y.-P., Yeh C.-Y., Huang P.-Y., Wang Z.-Y., Cheng H.-H., Li Y.-T., Chuang C.-F., Huang P.-C., Tang K.-T., Ma H.-P. (2015). A Battery-Less, Implantable Neuro-Electronic Interface for Studying the Mechanisms of Deep Brain Stimulation in Rat Models. IEEE Trans. Biomed. Circuits Syst..

[57] Wentz C.T., Bernstein J.G., Monahan P., Guerra A., Rodriguez A., Boyden E.S. (2011). A Wirelessly Powered and Controlled Device for Optical Neural Control of Freely-Behaving Animals. J. Neural Eng..

[58] Lazaro A., Boada M., Villarino R., Girbau D. (2019). Study on the Reading of Energy-Harvested Implanted NFC Tags Using Mobile Phones. IEEE Access.

[59] Ma S., Sydanheimo L., Ukkonen L., Bjorninen T. (2018). Split-Ring Resonator Antenna System with Cortical Implant and Head-Worn Parts for Effective Far-Field Implant Communications. IEEE Antennas Wirel. Propag. Lett..

[60] Miozzi C., Saggio G., Gruppioni E., Marrocco G. (2019). Constrained Safety-Integrity Performance of Through-the-Arms UHF-RFID Transcutaneous Wireless Communication for the Control of Prostheses. IEEE J. Radio Freq. Identif..

[61] Microsemi ZL70103 Medical Implantable RF Transceiver MICS-Band RF Telemetry, Datasheet, Revision 2. https://www.microsemi.com/product-directory/implantable-medical-transceivers/3915-zl70103#resources.

[62] Bae J., Song K., Lee H., Cho H., Yoo H.-J. (2011). A 0.24-nJ/b Wireless Body-Area-Network Transceiver with Scalable Double-FSK Modulation. IEEE J. Solid-State Circuits.

[63] Gao Y., Cheng S.-J., Toh W.-D., Kwok Y.-S., Tan K.-C.B., Chen X., Mok W.-M., Win H.-H., Zhao B., Diao S. (2013). An Asymmetrical QPSK/OOK Transceiver SoC and 15:1 JPEG Encoder IC for Multifunction Wireless Capsule Endoscopy. IEEE J. Solid-State Circuits.

[64] Liu Y.-H., Chen L.-G., Lin C.-Y., Lin T.-H. (2013). A 650-pJ/bit MedRadio Transmitter with an FIR-Embedded Phase Modulator for Medical Micro-Power Networks (MMNs). IEEE Trans. Circuits Syst. I: Regul. Pap..

[65] Cho H., Kim H., Kim M., Jang J., Lee Y., Lee K.J., Bae J., Yoo H.-J. (2015). A 79 pJ/b 80 Mb/s Full-Duplex Transceiver and a 100 kb/s Super-Regenerative Transceiver for Body Channel Communication. IEEE J. Solid-State Circuits.

[66] Saadeh W., Bin Altaf M.A., Alsuradi H., Yoo J. (2017). A Pseudo OFDM With Miniaturized FSK Demodulation Body-Coupled Communication Transceiver for Binaural Hearing Aids in 65 nm CMOS. IEEE J. Solid-State Circuits.

[67] Khaleghi A., Hasanvand A., Balasingham I. (2018). Radio Frequency Backscatter Communication for High Data Rate Deep Implants. IEEE Trans. Microw. Theory Tech..

